# CYCLOSPORINE IN CHRONIC IDIOPATHIC URTICARIA WITH POSITIVE AUTOLOGOUS SERUM SKIN TEST

**DOI:** 10.4103/0019-5154.41662

**Published:** 2008

**Authors:** K V Godse

**Affiliations:** *From Terna Hospital, Sector 10, Vashi, Navi Mumbai - 400 703, India. E-mail: drgodse@yahoo.co.in*

This small study evaluates the effectiveness and safety of cyclosporine (CsA) in the treatment of patients with chronic idiopathic urticaria with a positive autologous serum skin test (ASST), who fail to respond to conventional therapy, and requiring long-term oral steroid treatment. This small study evaluates the effectiveness and safety of cyclosporine (CsA) in the treatment of patients with chronic idiopathic urticaria with a positive autologous serum skin test (ASST), who fail to respond to conventional therapy, and requiring long-term oral steroid treatment. Small open-label trials suggest that cyclosporine (CsA), used at a range of different daily dosages (<5 mg/kg) and duration, could be of therapeutic value in patients with chronic idiopathic urticaria (CIU) that is unresponsive to conventional therapy.^1^–^4^

Five adult patients (4 females and 1 male) in the age group of 20-50 years with mean age of 37.8 years with severe disease ranging from 6 weeks to 5 years (Table 1), unresponsive to antihistamines and showing a positive ASST, were advised to take 3 mg/kg per day of CsA for 12 weeks after taking consent for treatment, along with cetirizine (10 mg). Exclusion criteria included other concomitant forms of urticaria, any contraindications to cetirizine and CsA, and relevant systemic disorders. The clinical efficacy was measured with an activity score of wheal numbers and itch (Urticaria Activity Score, UAS). All patients were followed up to assess response to treatment.

**Table 1:** Distribution of patients by sex and disease duration

Duration of urticaria	6 weeks to 1 year	More than 1 year
Male	0	1
Females	1	3

All patients were reviewed at 0,2,4,8 and 12 weeks with urticaria activity score.

The UAS consists of the sum of the wheal number score and the itch severity score.^5^ The wheal numbers are graded from 0 to 3 as follows: 0 - less than 10 small wheals (diameter, <3 cm); 1 - 10 to 50 small wheals or less than 10 large wheals (diameter, >3 cm); 2 - greater than 50 small wheals or 10 to 50 large wheals; and 3 - almost the whole body is covered. The severity of itching is graded from 0 to 3 (0, none; 1, mild; 2, moderate; and 3, severe). Baseline investigations included urine examination, complete blood count, blood sugar, urea, creatinine and serum electrolytes. Repeat tests were done at 2, 4, 8 and 12 weeks. Blood pressure was monitored every two weeks.

Average urticaria activity score was 5.4. Within two weeks of starting cyclosporine, the score came down to 1.6. The male patient discontinued cyclosporine due to high cost of therapy. Score came down to less than one in all four female patients who continued treatment. Side effects were few. In one patient, blood pressure went up 120/90 mm of Hg requiring amlodipine (2.5 mg) therapy. By the end of treatment, 3/4 (75%) patients were in full remission (score 0) and the remainder scored 1.

This uncontrolled study has shown that low-dose CsA is effective in treating CIU patients, and can be given safely for 3 months. One study showed that prolonged treatment with CsA is beneficial for maintaining remission in severe cases of CU. It spares the need for corticosteroids and is accompanied with mild side effects.^6^ We could not perform ASST after stopping treatment, but one study from Italy has shown that ASST becomes negative in majority of patients with remission of symptoms.^7^ Methotrexate can be used in patients who cannot afford cyclosporine as reported by us.^8^

## References

[1] Fradin MS, Ellis CN, Voorhes JJ (1991). Oral cyclosporine for severe chronic idiopathic urticaria and angioedema. J Am Acad Dermatol.

[2] Barlow RJ, Kobza BA, Greaves MW (1993). Treatment of severe, chronic urticaria with cyclosporine A. Eur J Dermatol.

[3] Toubi E, Blant A, Golan TD (1997). Low dose cyclosporine A in the treatment of severe chronic idiopathic urticaria. Allergy.

[4] Ilter N, Gurer MA, Akkoca MA (1999). Short term oral cyclosporine for chronic idiopathic urticaria. J Eur Acad Dermatol Venereol.

[5] Erbagci Z (2002). The leukotriene receptor antagonist montelukast in the treatment of chronic idiopathic urticaria: A single-blind, placebo-controlled, crossover clinical study. J Aller Clin Immunol.

[6] Kessel A, Toubi E (2006). Extended cyclosporine A treatment of severe chronic urticaria. Harefuah.

[7] Di Giacchino M, Di Stefano F, Cavallucci E, Verna N, Ramondo S, Paolini F (2003). Treatment of chronic idiopathic urticaria and positive autologous serum skin test with cyclosporine: Clinical and immunological evaluation. Allergy Asthma Proc.

[8] Godse K (2004). Methotrexate in autoimmune urticaria. Indian J Dermatol Venereol Leprol.

